# Routine childhood rabies pre-exposure prophylaxis can be cost effective in low- and middle-income countries

**DOI:** 10.1016/j.vaccine.2024.126703

**Published:** 2025-01-11

**Authors:** Adam John Ritchie, Aronrag Meeyai, Caroline Trotter, Alexander D. Douglas

**Affiliations:** a Jenner Institute, Old Road Campus Research Building, University of Oxford, Oxford OX3 7DQ, UK; b Centre for Tropical Medicine and Global Health, Nuffield Department of Medicine, University of Oxford, Oxford OX3 7LG, UK; c Department of Veterinary Medicine, University of Cambridge, Cambridge CB3 0ES, UK

**Keywords:** Rabies, Vaccination, Pre-exposure prophylaxis (PrEP), Cost-effectiveness, Modelling

## Abstract

**Background:**

Pre-exposure prophylactic rabies vaccination (PrEP) is advised for travellers to countries with high rabies incidence, but rarely available for local residents. Some studies suggest poor cost-effectiveness of PrEP in such settings, but have generally focused upon post-exposure prophylaxis (PEP) cost savings as the main benefit of PrEP, without considering lives saved by PrEP efficacy.

**Methods:**

We compared incremental cost-effectiveness ratios (ICERs) of use of rabies PrEP, against an alternative of using only PEP, by adapting a decision-tree model previously used to inform Gavi’s investment in rabies PEP. We consider scenarios including: a range of PrEP efficacies in individuals unable to access PEP; PrEP costs significantly below current prices (through single-dose approaches, inclusion in childhood vaccination schedules, increased manufacturing volume and/or new low-cost products); and variable rabies exposure risk and PEP access. We also present results from a simplified model, designed for ease of understanding.

**Results:**

Modelled ICERs were <1000 USD per quality adjusted life year (QALY) across a range of plausible combinations of rabies exposure risk, PEP access, PrEP cost and PrEP efficacy. If PrEP efficacy exceeds 50 % over 15 years, we estimate ICERs <500 USD/QALY where rabies incidence ≥3 per 100,000 per year and cost of vaccination is ≤5 USD/child. Under scenarios with lower rabies incidence of around 0.3 per 100,000 per year, due either to more limited exposure or greater access to PEP, ICERs <3000 USD may still be achieved even if PrEP efficacy is as low as 30 %.

**Conclusions:**

Routine childhood PrEP may be cost-effective in settings with modest willingness-to-pay, and rabies exposure risks plausible across much of Africa and South Asia. Cost-effectiveness requires low-cost PrEP regimes and some efficacy of PrEP in individuals unable to access PEP. Under such conditions, PrEP may be an attractive additional tool in the fight against rabies.

## 1 Introduction

Rabies is an entirely vaccine-preventable disease, yet results in tens of thousands of deaths each year. Improved access to post-exposure prophylaxis (PEP) and dog vaccination efforts are currently the key focus of rabies control policies [1–3]. Primarily due to cost, use of pre-exposure prophylaxis (PrEP) is only recommended in narrowly-defined populations where exposure risk is high, access to PEP limited, control in animal reservoirs difficult, and willingness-to-pay is relatively high [4,5]. Such populations include travellers from high-income countries and residents in the Peruvian Amazon [4–7]. In contrast, rabies disproportionately affects resource-limited settings where willingness-to-pay for health interventions is low.

Calculation of the cost-effectiveness of rabies control interventions is highly sensitive to estimates of the baseline burden of rabies disease. This is complicated by the fact that surveillance of disease and death are limited in the settings where rabies is most problematic, leading to significant under-reporting of rabies cases [4,5,7–9]. A widely-cited and in-depth modelling study of the global burden of rabies estimated 59,000 human deaths annually, with a 95 % confidence interval (CI) of 25,000–159,000 [9]. This corresponded to 3.7 million disability adjusted life years (DALYs) lost (95 % CI 1.6–10.4 million) and 8.6 billion USD of economic losses each year (95 % CI 2.9–21.5 billion) [9].

India is the country with the greatest number of rabies cases [8,9], although there are other countries where the risk per person is greater, especially in Africa [9]. In the above global modelling study, India was estimated to have 20,847 (95 % CI 7000–55,000) human rabies deaths per year [9]. A separate estimate of the number of furious rabies deaths in India in 2005 through analysis of death data in the Million Deaths Study suggested around 12,700 that year [8]. That is probably an underestimate as the verbal autopsy methodology used is unlikely to have been sensitive for paralytic and atypical rabies cases, which may represent 20–33 % of deaths [8]. Nonetheless it falls within the range of estimates for India from the 2015 Hampson et al. modelling study [9] and other sources [8].

Within India, analysis of the Million Death Study data reveals patterns similar to those reported elsewhere [6,10–14], with males, children and those living in rural areas at greatest risk [8]. Overall incidence of death from furious rabies across India was estimated to be 1.1/100,000 people per year. Among children aged 5–14 years, furious rabies accounted for over 1 % of all deaths, with those living in rural settings having a death rate of 2/100,000 children per year [8]. The study also identified substantial variability in risk between different regions, with incidence of ≥3.5/100,000 people per year in some states. This illustrates that cost-effectiveness of different rabies control policies – and hence the optimal choice of policy – may vary substantially sub-nationally.

Access to PEP in rabies-endemic areas is inconsistent and costly. Improved access to PEP could have a significant impact on human rabies deaths [15], and Gavi, the Vaccine Alliance, included support for this in their 2021–2025 vaccine investment strategy [3]. Following implementation delays due to the COVID-19 pandemic, Gavi opened a window for the first round of applications in July 2024. PEP is essentially an emergency treatment which should be administered as quickly as possible after a suspected rabies exposure [5]. Efficacy of PEP regimes without rabies immune globulin (RIG) may decrease after delays exceeding 24 hours [16]. Programmatic delivery of such a treatment is quite different from that for routine vaccinations [17]. The other major focus of rabies control is limiting exposure through elimination of dog rabies, with the Zero by 30 plan to end human deaths due to dog-mediated rabies by 2030 published by the WHO and others in 2018 [1,2]. It is estimated that for control of dog rabies, sustained vaccination coverage of at least 70 % of dogs is needed [5].

In most areas, PrEP has not been prioritised due to uncertainty over its cost-effectiveness. Results of some modelling studies suggest it is unlikely to be cost-effective in most rabies-endemic contexts [5,18], although others have provided conflicting analyses [10,12].

These differing conclusions have been contributed to by studies considering different – and in our view sometimes incomplete – potential benefits from PrEP. As compared to those who are rabies vaccine-naïve, people who have previously received PrEP require fewer doses of PEP and do not require rabies immunoglobulin (RIG) following an exposure [5,19]. Some models consider savings in PEP and RIG costs post-exposure as the primary benefit of PrEP [10,12,18]. A subset of studies have considered lives saved because PrEP makes adequate PEP more likely (with fewer doses needed and a longer time window in which it can be delivered after a bite) [10,12]. To our knowledge, no study has considered the possibility of benefits (lives saved) among PrEP recipients who are unable to access any PEP after an exposure.

Although PrEP without PEP should never be recommended, or a goal of policy, ongoing exposure through dog or other animal bites and inability to access timely PEP is unfortunately likely to remain a common scenario in some contexts. It therefore seems important to consider the possibility of PrEP efficacy in such cases. Reliance on virus neutralising antibody (VNA) titers exceeding 0.5 IU/mL as a surrogate of clinical protection may be partially responsible for the field’s tendency to disregard the possibility of clinical efficacy of PrEP alone: VNA titers can fall below this threshold relatively rapidly after some PrEP regimes [20–22]. Clinical protection may however be considerably more durable, due to anamnestic (memory / recall) responses upon exposure. We review relevant evidence below (Section 2.5). Notably, decreases in rabies deaths following PrEP deployment in the Peruvian Amazon [6,7,23] and the Philippines [24] are suggestive of substantial real world efficacy.

Durable human rabies elimination through dog vaccination and prompt PEP availability is clearly a ‘first choice’ approach. Universal PrEP would never be cost effective in a setting where this was achieved. We wished to consider, however, whether PrEP may have value as an ‘interim’ or ‘belt and braces’ approach, in settings where near-term feasibility of sustained elimination is uncertain, and considering recent and possible future changes in PrEP approaches.

Here, we model the marginal cost-effectiveness of rabies PrEP as compared to ‘no PrEP’ (ie: ‘PEP-only’) strategies, with two major changes from previous PrEP cost-effectiveness evaluations. Firstly, we consider a wider range of costs for delivering rabies PrEP, including the possibility of substantially lower costs. Costs could be lowered by larger volume production [25] (as compared to the status quo, under which human rabies vaccines are relatively low volume niche products) and/or by deploying a single dose approach to PrEP, either with existing or next generation vaccines [22,26]. Secondly, we model a range of efficacies for PrEP in preventing rabies in those who do not access PEP at all, or have incomplete or delayed access to PEP following exposure to rabies.

## 2 Methods

### 2.1 Assumptions used throughout

We present two models of marginal cost-effectiveness of PrEP as compared to an alternative scenario without PrEP (discussed further below), in contexts where human rabies elimination has not yet been achieved. Firstly, we present a simplified model, easily understandable by non-specialists, and influenced by only 3 input variables. Secondly, a decision tree model allows for a deeper analysis of a greater number of variables influencing cost effectiveness, and is structurally similar to models used in other evaluations of rabies vaccine cost-effectiveness [10,12,15].

For both models we examined benefit over a 15-year period after vaccination. We envisage inclusion of PrEP in childhood immunization schedules at c. 12 months of age. Rabies exposure risk varies across age groups and is highest in childhood [8,10–12,27].

We estimated that 50 QALYs would be gained per death averted. Rabies is a fatal disease for which there is no survival with disability. Gain of QALYs through rabies prevention is thus entirely due to averting premature death, with each year of perfect health gained worth 1 QALY. It was assumed that the risk of rabies death was consistent across the time horizon of the models, so that the average age of death was 8.5 years. World Bank data for 2021 showed only 7 countries (Lesotho, Chad, Nigeria, Central African Republic, Somalia, Eswatini and South Sudan) had a life expectancy at birth under 58.5 [28]. Even these countries would have higher life expectancy at age 8.5, which is beyond the age of peak infant and child mortality.

Although use of current PrEP schedules means cheaper PEP through fewer doses [5,19], our models assumed identical PEP utilisation and cost in those who have and have not received PrEP. We made this assumption as lower PrEP costs used in the models include a single-dose PrEP schedule, which may result in no changes to the recommended PEP schedule post-exposure. We also omitted costs of care for patients with rabies. These decisions are conservative in that including them would tend to increase the apparent marginal cost-effectiveness of the PrEP-based strategy.

The models make no assumptions about what animal is the source of potential rabies exposures through bites, although most of the information informing these parameters in the decision tree model are primarily related to dog bites.

For both models, there was a designated ‘base case’ analysis using defined values for each variable input parameter.

### 2.2 Structure of simplified model

A simplified, static model was built in *MS Excel* (see Supplementary Material).

Input variables were;

- baseline rabies burden in a ‘no PrEP’ comparator scenario, measured as rabies deaths per year per 100,000 children;
- PrEP efficacy, measured as the proportion of deaths in the ‘no PrEP’ scenario averted by PrEP during the 15 years post-vaccination. This encompasses both protection in the absence of any PEP and enhanced efficacy of delayed or incomplete PEP;
- total cost of delivery of a complete course of PrEP per child, in USD.

Output variables were:

- number needed to vaccinate (NNV) to save one life in the following 15 years [29]. Any possible effect upon deaths more than 15 years after vaccination was ignored.
- cost per life saved;
- cost per QALY gained.

The formulae used to calculate the outputs were;

- control event rate (CER) = rabies burden × childhood years
- experimental event rate (EER) = CER × (1-PrEP efficacy)
- absolute risk reduction (ARR) CER – EER
- NNV = 1/ARR
- cost per life saved = cost of PrEP per child ×NNV
- cost per QALY gained = cost per life saved / QALYs saved per death averted

### 2.3 Simplified model parameter values

Rabies burden is the number of deaths from rabies per 100,000 children per year. We used a central value of 1, which has been estimated to be the average (all-age) rabies mortality rate in countries representing over 25 % of the global population [9] and similar to the overall furious rabies death rate across India estimated by analysis of the Million Deaths study [8]. Incidence in children is believed to be higher than in adults. We used a lower value of 0.3 and an upper value of 3, which are the estimated rate of deaths from rabies in countries representing over 50 % and 6 % of the global population respectively [9] and similar to rates reported for low and high burden states in India [8].

PrEP efficacy represents mean efficacy against rabies death over 15 years, encompassing both deaths averted among individuals who receive no PEP, and deaths averted among individuals receiving ineffective / inadequate PEP. We used values of 0.8, 0.6 and 0.3. Justification for PrEP efficacy is discussed further in the Supplementary Methods.

We used a single estimate for the total incremental cost of delivered PrEP as part of existing routine childhood immunization schedules (as opposed to costs per dose). This total includes cost of goods and programmatic costs of distribution and administration, with values of 2 and 5 USD used. Based upon comprehensive analysis of vaccine programme costs performed by the International Vaccine Access Center, [30] we believe these to be plausible incremental costs if a novel single dose PrEP is co-administered with a vaccine already included in routine childhood immunization programmes, and possibly also achievable if doses of a low-cost two-dose regime are administered at two such visits.

As an overall ‘base case’ for the simplified model, we used a cost of PrEP of USD 5, and values of rabies burden and PrEP efficacy corresponding to central values used in the decision tree model, as explained further below.

### 2.4 Structure of decision tree model

A decision-tree model was used to represent events and their consequences (costs and outcomes) for human rabies. The model was adapted from the probability decision-tree framework developed by the WHO Rabies Modelling Consortium [15], and similar in approach to other models used to estimate the health and economic impacts of rabies PrEP [10,12]. It was built using **R** language and can be accessed at https://github.com/HealthDecisionAnalyst/PrEP-rabies.

**Fig. 1:**
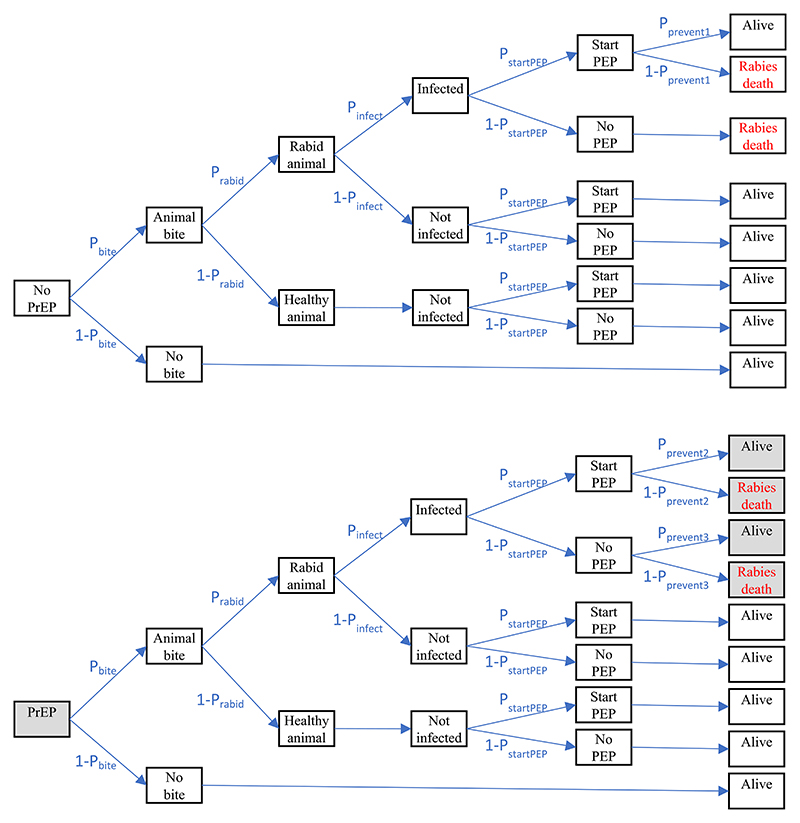
The decision tree model comparing the PEP-only (top half) to the PrEP plus PEP (bottom half) scenarios. Differences in the PrEP plus PEP scenario, which are cost of PrEP, the probability of surviving a rabies exposure in the absence of PEP (P_prevent3_), and the probability of surviving a rabies exposure where timely PEP is started (P_prevent2_), are highlighted in grey.

The model was used to compare scenarios with and without routine childhood PrEP. Differences between the PrEP and non-PrEP scenarios are costs of PrEP, the probability of surviving a rabies exposure in the absence of PEP, and the probability of surviving a rabies exposure where PEP is started on time to prevent rabies (which includes the possibility of the multi-dose PEP schedule not being completed in the non-PrEP scenario) (Fig. 1).

The output of the decision tree model is the incremental cost-effectiveness ratio (ICER), equivalent to the cost in USD per QALY gained due to rabies deaths being avoided.

For key input parameters that may vary in different settings or where there is uncertainty over their true value, a series of sensitivity analyses were carried out. Ranges of parameter values were chosen to reflect plausible values in real-world low- and middle-income country contexts which do not have robust dog vaccination programmes, where human rabies is a significant public health problem and where access to emergency healthcare, even for more common infections, is imperfect.

For one-way sensitivity analysis, all values except for one were fixed and then the impact of varying that one value on the ICER quantified. The fixed values and ranges for key parameters are discussed below and shown in Table 1.

For multi-variate sensitivity analysis, five different values for each of four key parameters were used, as outlined in Table 1.

### 2.5 Decision tree model input parameter values

Further details and justifications of the input parameter values are available in the Supplementary Methods.

#### 2.5.1 Cost of PrEP

We used a single estimate for the total incremental cost of delivered PrEP that includes cost of goods and programmatic costs of distribution and administration. We used a base case value of USD 5, a lower limit of USD 2, and upper limit of USD 45.

The upper end of the range was used to include costs of multi-visit schedules currently used for PrEP The lower end of the range reflects the possibility of single-dose PrEP, preferably co-administered at an existing EPI visit.

#### 2.5.2 Probability of being bitten (P_bite_)

We considered only high-risk bites from suspected, probably, or confirmed rabid animals which would be categorised by WHO guidance as class II-III, i.e. for which PEP would be indicated [5].

For annual probability of such a high-risk bite (P_bite_), we used a base case value of 0.001 (100 per 100,000 per year), a lower limit of 0.0001 (10/100,000), and an upper limit of 0.01 (1000/100,000).

#### 2.5.3 Probability biting animal is rabid (P_rabid_)

Again, only WHO class II-III exposures were considered.

We used a base case value of 0.3, a lower limit of 0.1, and an upper limit of 0.86.

#### 2.5.4 Probability of being infected with rabies if bitten by a rabid animal (Pinfect)

This excludes the impact of PrEP or PEP, which are incorporated in other parts of the model.

A fixed value of 0.19 was used.

#### 2.5.5 Rabies exposure risk per 100,000 people

This was used for the multi-variate sensitivity analysis only. It is the number of rabies cases expected per 100,000 people per year in the absence of any vaccination or treatment. It is the multiplicative product of P_bite_, P_rabid_ and P_infect_, to give the annual probability per person per year, multiplied by 100,000. Our base case scenario values of these parameters result in estimated rabies exposure risk, in the absence of any human rabies vaccination or treatment, of 5.7 per 100,000 per year.

#### 2.5.6 Probability of starting timely PEP (P_startPEP_)

This is the probability that someone who was exposed to rabies starts PEP quickly enough for it to prevent rabies if all required PEP doses are completed.

We used a base case value of 0.5, a lower limit of 0.1 and an upper limit of 0.9.

#### 2.5.7 Probability PEP prevents rabies in absence of PrEP (P_prevent1_)

This is the efficacy of PEP given to a rabies-vaccine-naïve recipient (i.e. the probability that a PrEP-naïve bite recipient, who would otherwise have developed rabies, instead does not develop rabies due to starting timely PEP). For simplicity, this includes a mixture of individuals receiving timely and complete PEP and individuals receiving timely but incomplete PEP.

We used a base case value of 0.94, a lower limit of 0.89 and an upper limit of 0.99.

#### 2.5.8 Probability that PEP and/or PrEP prevent rabies when both are received (P_prevent2_)

This is the probability of someone who would have developed rabies but had previously received PrEP *and* started timely PEP then not developing rabies (which may be due to PrEP, PEP or the combination of both).

A fixed value of 1.0 was used.

#### 2.5.9 Probability PrEP prevents rabies in absence of PEP (P_prevent3_)

This represents mean PrEP vaccine efficacy against death over 15 years, among individuals not accessing any PEP.

We used a base case value of 0.6, a lower limit of 0.0 and an upper limit of 0.95.

Uncertainty about the appropriate value for this parameter is both high and important for the conclusions of the present analysis. For reasons discussed in the Supplementary Methods, we used a very broad range of values in our sensitivity analyses, spanning from what we would consider to be an implausibly low lower bound (0.0, i.e. no efficacy) to a very high upper bound (0.95).

#### 2.5.10 Discount rate

Discount rates of 0 %, 1.5 % and 3 % per annum were applied to the value of future health in the decision tree model. The main results reported in this paper are for a 0 % discount rate, with the effect of 1.5 % and 3 % discount rates on the ICER range in one-way sensitivity analyses and multi-variable contour plots show in Supplementary Figs. 1, 2 and 3.

### 2.6 Calculation of parameters for simplified model ‘base case’

Central values of decision-tree model parameters, as stated above (Table 1), were used to calculate simplified model parameters for a ‘base case’ and hence to directly compare the output of the two models. For this comparison, cost of PrEP was assumed to be USD 5 for both models. Further details are provided in the Supplementary Methods.

## 3 Results

### 3.1 Base case scenario

We applied both simplified and decision-tree models (in Excel and R respectively) to estimate cost-effectiveness of PrEP under a ‘base case’ scenario.

**Fig. 2:**
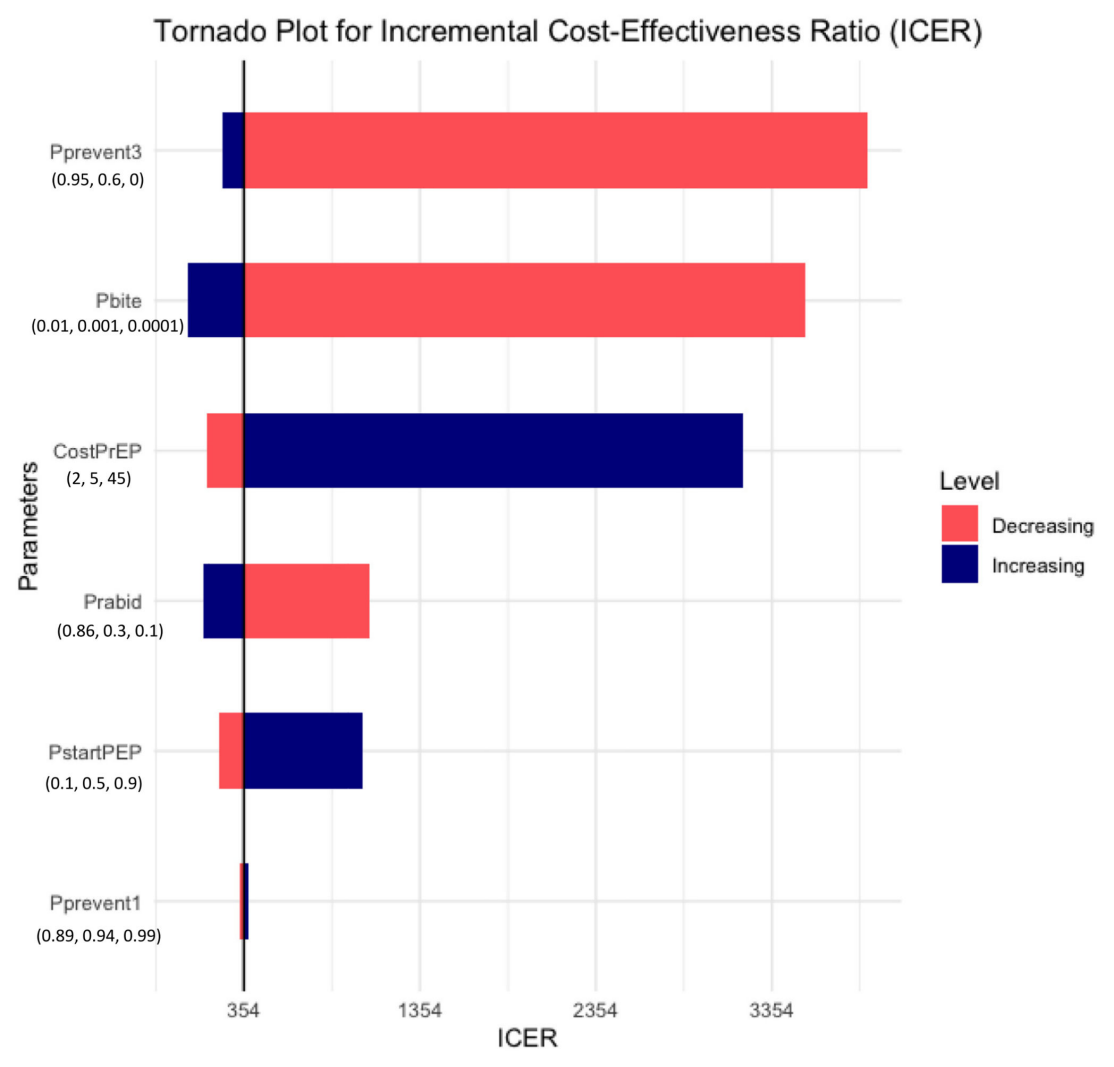
Tornado plot of one-way sensitivity analysis of key parameters using the decision tree model when no discount rate was applied. Parameter values used are outlined in Table 1: values of one parameter at a time (indicated on the y axis) were varied within the stated range, while all other parameters were fixed at the base case values. Below each variable label, the value of that variable generating the lowest, base case, and maximum ICERs are listed. The effect of decreasing and increasing each parameter value on the ICER are indicated by the pink and blue coloured bars respectively. The black vertical line indicates the base case scenario, which gave an ICER of 354 USD/QALY. (For interpretation of the references to colour in this figure legend, the reader is referred to the web version of this article.)

For the simplified model, parameter values were calculated from ‘base case’ values of decision tree model parameters as set out in Table 1 and Section 2.6). Both indicated a cost of 354 USD per QALY gained (Table 2, Fig. 2).

When a discount rate of 1.5 % or 3 % was applied, the ICER increased to 506 USD or 688 USD respectively (Supplementary Fig. 1).

### 3.2 Simplified model: sensitivity to input parameters

The simplified model included only three variables which we expected to substantially influence cost per QALY gained: baseline rabies burden; PrEP efficacy; and cost of PrEP. Sensitivity analyses varying the parameter values in this simplified model suggested cost per QALY ranging from 111 to 7408 USD (Table 2, Supplementary Material). All three input variables had a meaningful impact on the output variables.

### 3.3 Decision tree model: One-way sensitivity analysis

Using the more complex decision tree model, we were able to explore the impact of changes in key variables on cost effectiveness in greater detail. As compared to the simplified model, this included a further breakdown of rabies burden into various components of risk of rabies exposure, such as the probabilities of being bitten by an animal and of that animal having rabies. Similarly, the impact of PrEP on survival following a subsequent rabies exposure was broken down into the probability of PrEP preventing a case of rabies in the absence of PEP (P_prevent3_), and the impact of previously receiving PrEP on the probability of surviving if PEP was initiated within 2-weeks post-exposure (the difference between P_prevent2_ and P_prevent1_).

In this model, P_bite_, P_rabid_, P_infect_, P_startPEP_, and P_prevent1_ define the background risk of death due to rabies in the ‘no PrEP’ scenario. This background risk of rabies in the ‘no PrEP’ scenario is effectively the pool of lives which PrEP may save. Cost-effectiveness of PrEP is thus expected to be proportional to this risk.

Under the base case estimates of P_startPEP_ = 0.5 and PEP efficacy in the absence of PrEP (i.e. P_prevent1_) = 0.94, most deaths will occur in individuals who do not receive any PEP. Generally, there are expected to be a substantially smaller number of deaths among those who *do* receive PEP (mostly among those for whom it is incomplete or late), and relatively little of the benefit of PrEP would be expected to be among this group. As expected, then, variation of P_bite_, P_rabid_ and P_startPEP_ across the plausible ranges was estimated to have a substantial effect upon estimated cost-effectiveness of PrEP, while variation in PEP efficacy in the absence of PrEP (P_prevent_1) was found to have relatively little effect (Fig. 2). There was no variation in P_infect_, which was assigned a fixed value.

Variation across the ranges of PrEP efficacy in the absence of PEP (P_prevent3_) and the cost of PrEP also substantially affected cost-effectiveness (Fig. 2).

**Fig. 3:**
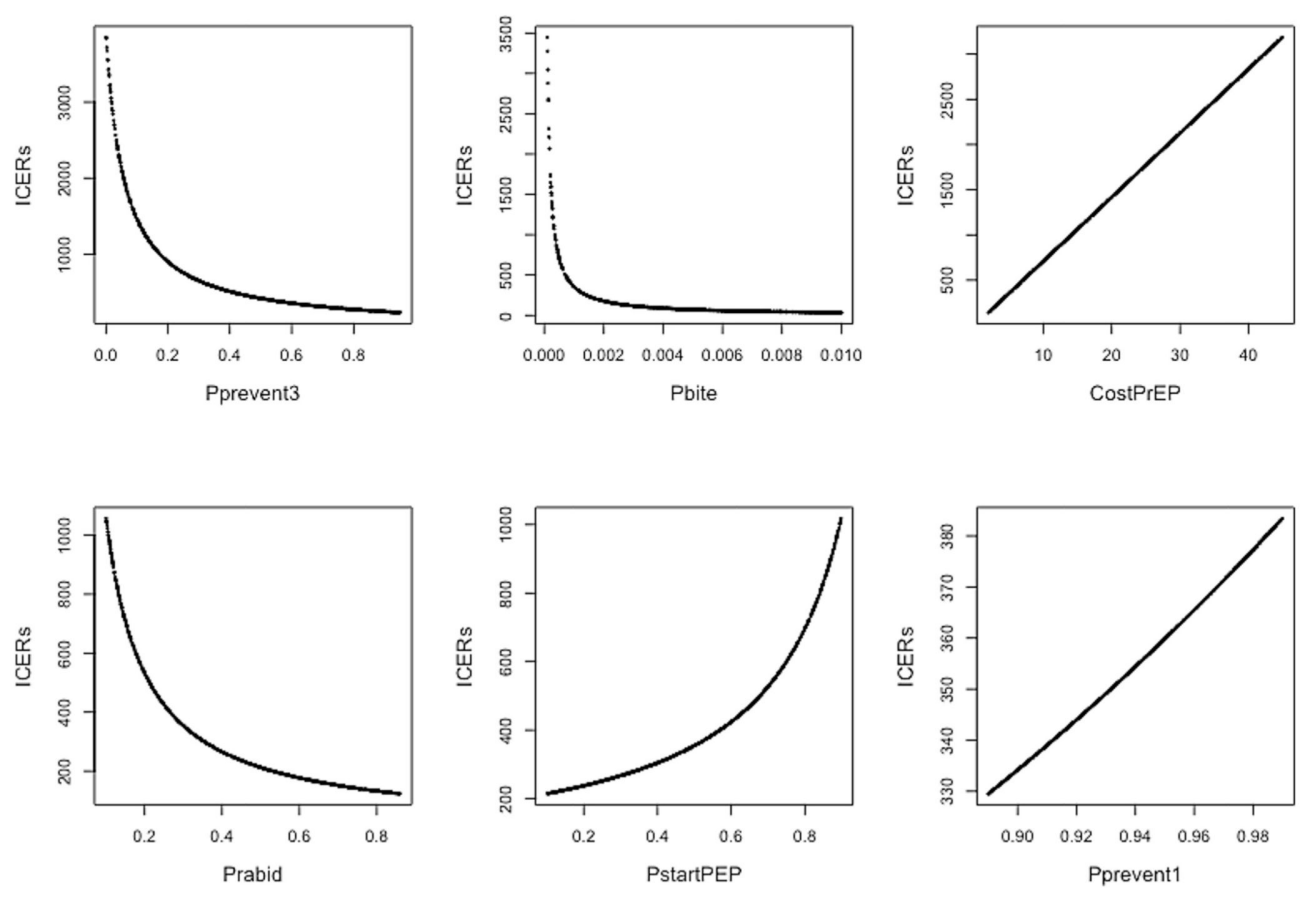
Individual plots of the impact of varying each key parameter in a one-way sensitivity analysis. Values of the parameter indicated were varied on the x axis within a range and values of other parameter values fixed as outlined in Table 1. Note the scale of the y axis (ICERs) varies between each plot.

Variation in P_prevent3_ had the largest effect on the ICER: this is partly because PrEP efficacy in the absence of PEP is potentially the main route of benefit of PrEP, but it is largely a result of the wide range of values of this parameter which we explored (from 0 to 0.95), due to the high degree of uncertainty. As expected, falling PrEP efficacy was associated with a steeply rising ICER but notably the ICER remained <1000 USD/QALY unless P_prevent3_ was <0.2 (Fig. 3).

### 3.4 Multi-variate sensitivity analysis

**Fig. 4:**
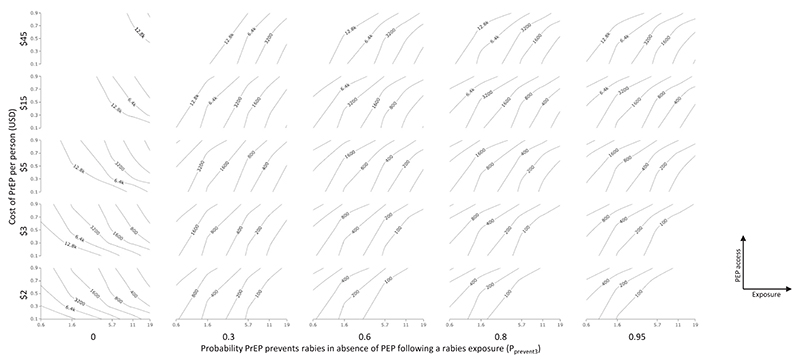
Multi-variate sensitivity analysis, represented as contour plots of the relationship of the ICER with combinations of four variables. In the grid of 5 × 5 individual contour plots, the probability P_prevent3_ (efficacy of PrEP in absence of PEP) varies between the columns as shown on the outer X-axis, with 0 for the left column and 0.95 for the right column. Rabies PrEP cost varies between the rows as shown on the outer Y-axis, with 45 USD for the top row and 2 USD for the bottom row. For each individual contour plot, rabies exposure risk (Exposure) per 100,000 people (made up of *P*_bite_ × *P*_rabid_ × *P*_infect_ × 100,000) varies across the X axis from 0.56 to 19, while access to PEP (measured as the probability of starting PEP following a rabies exposure, or P_startPEP_) varies on the Y axis from 0.1 to 0.9. Poor access to PEP (inner Y axis, low values), higher levels of rabies risk (inner x axis, high values), lower cost of PrEP (outer y axis, low values) and higher efficacy of PrEP in preventing rabies in the absence of PEP (outer x axis, high values) all lower the ICER for PrEP.

Multi-factor sensitivity analysis showed that ICERs of under USD 3000 per QALY gained are realistic across a wide range of scenarios that are likely reflective of real-world scenarios, even if PrEP had a probability of preventing rabies in the absence of PEP of 0.3 (Fig. 4, Supplementary Fig. 3).

As would be expected, higher levels of PrEP efficacy (P_prevent3_) and lower cost of PrEP result in lower ICERs. When P_prevent3_ > 0, cost-effectiveness of PrEP is more favourable, for a given rate of rabies exposures, when access to PEP is less good (which would be expected to result in more deaths in the PEP-only scenario).

In contrast, in the scenario under which PrEP alone has no efficacy (P_prevent3_ = 0, shown in the left-hand column of panels in Fig. 4), cost effectiveness of PrEP is generally poor but (unlike the other scenarios) is improved by *better* access to PEP. This is because, under this scenario, PrEP benefit is only possible through the mechanism of improved efficacy of late / delayed PEP. This is the scenario that has been considered in some previous modelling of rabies PrEP cost effectiveness.

## 4 Discussion

Our models demonstrate that the use of rabies PrEP as a public health intervention could save lives and be cost-effective in a range of plausible scenarios. Despite efforts to increase the use of PEP and/or rabies control in dogs [1–3,15], there are some settings where it is unclear when canine rabies elimination and universal PEP access will be achieved. Our analysis suggests that lower-cost PrEP regimes (whether with new or existing vaccines, as part of routine childhood vaccination and ideally using new combination products) would substantially enhance PrEP cost-efficacy, such that it offers a valuable interim public health measure in such settings.

Governments and funders should not view PrEP as an alternative to PEP or dog vaccination, but nor should it continue to be viewed as a tool to be reserved only for those with the highest willingness to pay. Instead, we suggest PrEP could be considered a ‘belt and braces’ in settings where childhood vaccination programmes exist but rabies incidence is high and frank analysis suggests uncertainty about feasibility of near-term canine rabies elimination.

The key differences between our work and previous assessments of PrEP cost effectiveness are that we have considered benefit of PrEP in terms of lives saved (rather than simply cost savings), and that we have considered lower costs of delivering PrEP (as would be expected if vaccine was manufactured at greater scale, delivered at a single visit, and/or co-administered with existing EPI vaccines) [17,22,25,37,39,46]. Most previous models have focused on savings from decreased costs of PEP and/or treatment of rabies cases as the economic benefit of multi-dose PrEP [10,12,18]. While some papers have considered lives save through PrEP, this has been due to PrEP improving PEP efficacy [10,12], and our study is the first to consider the efficacy in PrEP in the absence of PEP. It is clear that ignoring this potential effect of PrEP decreases its apparent cost-effectiveness, and also leads to a greatly altered interactive effect between the cost of PrEP and rabies exposure risk in relation to the ICER of PrEP (Fig. 4). It is our view that previous modelling studies generally have not fully considered incremental efficacy which may be achieved by PrEP, i.e. the possibility that policies including PrEP might cost more than PEP-only policies, yet this additional cost could nonetheless represent a worthwhile investment. This would be the case if policies including PrEP averted more deaths due to rabies than PEP-only policies, and did so with incremental cost per QALY year gained which fell under local willingness-to-pay thresholds.

Rabies deaths occur in cases where PEP is either not sought, is inaccessible, delayed or incomplete [27,37,47]. It is likely that PrEP could offer significant benefits in terms of lives saved in such scenarios, with duration of clinical efficacy probably exceeding the duration of maintenance of VNA >0.5 IU/mL (Section 2.5) [24,37]. Benefit of PrEP in the absence of prompt (or any) PEP is of course the justification for its administration to high-income-country travellers to high risk rabies endemic areas (in WHO’s wording, places where “access to adequate PEP is not guaranteed”) [5], and indeed the basis of animal rabies vaccination (PEP in animals is rare in most LMIC settings).The fact PrEP is not available for children who live permanently in those same places is starkly inequitable [24].

Estimating or measuring the efficacy of rabies PrEP in humans is difficult, especially at longer time intervals after vaccination. A broad range of values are plausible, as described in the methods section of this study. Further evaluation of long-term efficacy in large populations which have received PrEP, for example in Peru and the Philippines, would be highly worthwhile. Ultimately however the true long-term efficacy of PrEP may only become known if a large (country-wide or state-wide) vaccination campaign is rolled out in conjunction with a carefully designed large-scale real-world evaluation of efficacy (e.g. using test-negative designs or incidence in comparator groups initially ineligible for vaccination to infer efficacy). Our study shows that variation of PrEP efficacy within the current range of uncertainty would have a major effect upon cost-effectiveness (Fig. 2, Table 2) and that at efficacy levels above 20 % over 15 years, PrEP cost-effectiveness improves steeply (Fig. 3, Fig. 4). These findings support the value of real-world evaluation of PrEP efficacy: in the absence of such data, there is a risk that PrEP efficacy and hence cost-effectiveness are seriously under-estimated.

Rabies vaccines remain relatively expensive and are often not available when needed. Single-dose approaches to PrEP offer lower overall costs, higher vaccination rates and easier integration into childhood vaccination schedules than current, multi-dose schedules [24,48,49]. Alternatively, doses of current inactivated virus rabies antigen could be included in new combination vaccines, for example pentavalent or hexavalent products. Such products already include both inactivated polio virus (a similar immunogen to rabies vaccine) and tetanus toxoid. Tetanus offers a precedent for a ‘routine PrEP for all, emergency PEP if needed’ approach. Inclusion of rabies vaccination in childhood vaccination schedules through either of these approaches would increase and smooth demand, signalling a return on investment for manufacturers, lowering cost per dose, and improving availability of doses for both PrEP and PEP.

Both factors specific to PrEP (such as efficacy and price) and factors beyond PrEP (such as PEP access and rabies exposure risk) influence its cost effectiveness. PrEP will be most cost effective where there is high risk of rabies exposure and poor access to rabies PEP. These two important factors are the focus of public health programmes to increase access to PEP and decrease the exposure risk through dog vaccination. Recent analysis suggests rabies elimination through dog vaccination, although possible and highly desirable, may unfortunately be more challenging in some contexts than previously thought [50]. There is likely to be large variation of rabies exposure risk and access to PEP within countries, especially between urban and rural settings. Rabies PrEP policy could vary accordingly at sub-national level, with the case for routine PrEP likely to be stronger in rural areas.

Where rabies exposure can be virtually eliminated or effective treatment through PEP made universally available, there is no need for PrEP. With half the global population living in countries with significant lifetime risk of rabies and around 60,000 deaths per year, it is clear however that these conditions have not been met for far too many people [9]. The use of PrEP in such contexts, depending on the cost and efficacy of PrEP, has the potential to save many of these lives in a way likely to be cost-effective (Table 2, Fig. 4). PrEP should be seen as another tool available to fight rabies where needed, as a complement to PEP and dog vaccination programmes.

## Supplementary Material

**Appendix A.Supplementary data**

Supplementary data to this article can be found online at https://doi.org/10.1016/j.vaccine.2024.126703.

Supp file

Supp file

## Figures and Tables

**Table 1:** Decision tree model input parameters.

Parameters that vary	Fixed values for base case analysis	Range for one-way sensitivity analysis (tornado plot)	Values for multi-variate sensitivity analysis (contour plots)	Sources
Cost of PrEP (USD)	5	2–45	2, 3, 5, 15, 45	[10,12,18,25,31]
P_bite_	0.001	0.0001–0.01	0.0001, 0.0001, 0.001, 0.003, 0.01	[9,10,16,18,27,32,33]
P_rabid_	0.3	0.1–0.86	0.3, 0.86, 0.3, 0.2, 0.1	[9,11,12,15,16,18,27,32–34]
P_startPEP_	0.5	0.1–0.9	0.1, 0.3, 0.5, 0.7, 0.9	[11,13,15,16,27,32]
P_prevent1_	0.94	0.89–0.99	0.94 (fixed)	[16]
P_prevent3_	0.6	0.0–0.95	0.0, 0.3, 0.6, 0.8, 0.95	[6,7,19–21,24,35–45]
Rabies exposure risk per 100,000 people*	5.7	0.57–19.0	0.57, 1.63, 5.7, 11.4, 19.0	NA
Parameters that are fixed		Fixed values		Source
P_infect_		0.19		[10–12,16,18]
P_prevent2_		1.0		[5,19]
Childhood years		15		[8,10–12,27]
QALYS gained per rabies death averted		50		[28]

* Rabies exposure risk per 100,000 people is not an independent variable. It represents the number of rabies cases expected in a population of 100,000 people per year in the absence of any vaccination or treatment. It is the multiplicative product of P_bite_, P_rabid_ and P_infect_, to give the annual probability per person per year, multiplied by 100,000.

**Table 2:** Variable inputs and cost per QALY gained for the simplified model, including the base case scenario.

																			Base case
Cost of PrEP (USD)	5	5	5	5	5	5	5	5	5	2	2	2	2	2	2	2	2	2	5
Rabies burden	3	3	3	1	1	1	0.3	0.3	0.3	3	3	3	1	1	1	0.3	0.3	0.3	3.021
PrEP efficacy	0.8	0.6	0.3	0.8	0.6	0.3	0.8	0.6	0.3	0.8	0.6	0.3	0.8	0.6	0.3	0.8	0.6	0.3	0.623
Cost/QALY gained (USD)	278	370	741	833	1111	2222	2778	3704	7408	111	148	296	333	444	889	1111	1482	2963	354

## Data Availability

Model data/code is available for download as part of Supplementary Materials and via the GitHub link provided in the paper.
