# Supplementary material for: Routine childhood rabies pre-exposure prophylaxis can be cost effective in low- and middle-income countries
Source: Vaccine. Author manuscript; Available in PMC 2025 Sep 27. (PMC7618176; doi:10.1016/j.vaccine.2024.126703)
Supplement: Supp file [file EMS208571-supplement-Supp_file.docx]

Supplementary Material

# Supplementary Methods

## Decision tree model input parameter values

#### Cost of PrEP

We used a single estimate for the total incremental cost of delivered PrEP that includes cost of goods and programmatic costs of distribution and administration. We used a base case value of USD 5, a lower limit of USD 2, and upper limit of USD 45.

The upper end of the range was used to include costs of multi-visit schedules currently used for PrEP The lower end of the range reflects the possibility of single-dose PrEP, preferably co-administered at an existing EPI visit.

Other studies have estimated costs for two or three dose PrEP in the range USD 5-83 [1-3]. The WHO Global Vaccine Market Report 2022 gives costs per dose of rabies vaccine for middle income countries of USD 9 to USD 10 in 2021 [4]. Greater use of PrEP and large-volume purchase agreements would be likely to result in substantial economies of scale [5].

#### Probability of being bitten (P_bite_)

Estimates of bite probability and probability a biting animal is rabid are both influenced by the definition of a bite used, with the two being inversely related: increasingly stringent definitions of high-risk bites tend to result in lower annual bite probability and higher probability the biting animal is rabid.

We considered only high-risk bites from suspected, probably, or confirmed rabid animals which would be categorised by WHO guidance as class II-III, i.e. for which PEP would be indicated [6].

For annual probability of such a high-risk bite (P_bite_), we used a base case value of 0.001 (100 per 100 000 per year), a lower limit of 0.0001 (10/100 000), and an upper limit of 0.01 (1000/100 000).

Most field studies from areas regarded as having problematic rabies burden report bite incidences in, or close to, this range [2, 7-10]. Hampson *et al* analyses of the global burden of rabies [11] and modelling of prophylaxis [1] used similar ranges.

Some studies have reported higher bite incidence rates. Such studies generally used broader case definitions including lower-risk exposures [3, 12-14].

#### Probability biting animal is rabid (P_rabid_)

Again, only WHO class II-III exposures were considered.

We used a base case value of 0.3, a lower limit of 0.1, and an upper limit of 0.86.

Rates within this range were reported in several field studies [7-10, 15, 16] and similar values have been used by a number of other model-based studies [1, 3, 11, 14].

A lower value of 0.022 used in one modelling study was not included, as the source data could not be fully identified and it was not clear that the case definition used to identify biting animals was linked to high-risk exposures [2].

#### Probability of being infected with rabies if bitten by a rabid animal (P_infect_)

This excludes the impact of PrEP or PEP, which are incorporated in other parts of the model.

A fixed value of 0.19 was used. This is based on several studies suggesting this (or similar) rates as the real world risk of infection with rabies following a bite by a rabid animal [7, 16] and this rate being used in several other models as the risk following category II/III exposures [1-3].

#### Rabies exposure risk per 100,000 people

This was used for the multi-variate sensitivity analysis only. It is the number of rabies cases expected per 100 000 people per year in the absence of any vaccination or treatment. It is the multiplicative product of P_bite_, P_rabid_ and P_infect_, to give the annual probability per person per year, multiplied by 100 000. Our base case scenario values of these parameters result in estimated rabies exposure risk, in the absence of any human rabies vaccination or treatment, of 5.7 per 100,000 per year. This number will usually be higher than the rabies burden in a given area (which is used in the simplified model), due to access to PEP lowering the number of actual cases. Although the full range of *P_bite_* and P*_rabid_* values are plausible based on the literature, differences in definitions of bite mean values at the upper ends of the plausible ranges of both (0.01 and 0.86 respectively) are unlikely to occur together. If they did, they would give a rabies exposure risk of over 160 cases per 100 000 people per year, which is implausibly high. Thus, in the multi-variate analysis, values of *P_bite_* and P*_rabid_* were used to give a high, but not entirely implausible upper limit of 19 exposures per 100 000 people per year in the absence of any PrEP or PEP (Table 1).

#### Probability of starting timely PEP (P_startPEP_)

This is the probability that someone who was exposed to rabies starts PEP quickly enough for it to prevent rabies if all required PEP doses are completed. How quickly is ‘quickly enough’ will vary according to individual circumstances (for example the nature of the exposure) i.e. the parameter does not reflect vaccination within a certain fixed time period. The majority of rabies cases have an incubation period of 2-3 months, but for 2-3% of cases it is over a year [6]. The WHO recommends PEP is started immediately after exposure (where it has the highest efficacy), but that it can save lives even if initiated even after a delay due to the variable incubation period [6, 17].

This probability can vary greatly, and is strongly influenced by several factors, including geographical, economic, cultural, and health system context (in particular, access to healthcare facilities and availability of rabies vaccine at those facilities).

We used a base case value of 0.5, a lower limit of 0.1 and an upper limit of 0.9.

Studies of exposure and PEP in Tanzania and India have suggested probabilities of starting PEP in the range 0.63 to 0.86 [7, 8, 12, 16], while a study from Cambodia suggested a figure of 0.176 nationally with large variability between regions [9]. There are likely to be many less studied areas with poor access, and PEP-seeking behaviour can also vary greatly. The most comprehensive, recent modelling of PEP access included a range of 0.05 to 1.0 for the probability of seeking care when potentially exposed, coupled with a range of 0.05 to 0.98 for the probability of receiving PEP if treatment was sought [14].

#### Probability PEP prevents rabies in absence of PrEP (P_prevent1_)

This is the efficacy of PEP given to a rabies-vaccine-naïve recipient (i.e. the probability that a PrEP-naïve bite recipient, who would otherwise have developed rabies, instead does not develop rabies due to starting timely PEP). For simplicity, this includes a mixture of individuals receiving timely and complete PEP and individuals receiving timely but incomplete PEP.

We used a base case value of 0.94, a lower limit of 0.89 and an upper limit of 0.99.

Although there are intermittent case reports of failure [18-20], PEP is highly effective in preventing rabies when administration begins within 24 hours post-exposure and the appropriate multi-dose regimen is completed. PEP efficacy less than 1 in non-PrEP-recipients instead reflects the fact that not all doses are received by all who start PEP.

Although a figure of 0.986 has been used as the probability of incomplete/delayed PEP preventing rabies in some modelling [14], we believe this to be an overestimate. The source data for this estimate can be found in the study by Changalucha *et al*. of PEP in Tanzania, where 14 individuals were suspected of developing rabies, out of 1005 individuals who were potentially exposed but received incomplete/delayed PEP [7]. The 0.986 probability was thus the probability of not developing rabies after receiving incomplete/delayed PEP, but other data in the same paper suggests that the probability of not developing rabies with no PEP at all was 0.835. We therefor use an estimate of the efficacy of delayed / incomplete PEP of 0.916, as of the 1005 individuals only 166 would have been expected to get rabies in the absence of PEP.

Using the same study’s data for all individuals receiving any PEP (irrespective of timing and completeness and, as per the model to be rolled out by Gavi, without RIG), overall PEP efficacy in individuals who have not been previously vaccinated can be estimated as 0.94 [7].

It is worth noting that improved PEP access would be expected to increase both the probability of timely PEP initiation (P_StartPEP_) *and* the probability of PEP being complete, with the latter effect manifesting as increased PEP efficacy (P_prevent1_). Inclusion of both variables in the decision tree model, with the upper limit of PEP efficacy extending to 0.99, therefore allows both of these effects to be considered.

#### Probability that PEP and/or PrEP prevent rabies when both are received (P_prevent2_)

This is the probability of someone who would have developed rabies but had previously received PrEP *and* started timely PEP then not developing rabies (which may be due to PrEP, PEP or the combination of both).

A fixed value of 1.0 was used. This figure is higher than the range used for PEP efficacy in individuals who did not receive PrEP (P_prevent1_) because, for previous PrEP recipients, a single visit, 4-site, ID administration is considered a full course of PEP (i.e. there is not a risk of a course being started but not completed) [17].

#### Probability PrEP prevents rabies in absence of PEP (P_prevent3_)

This represents mean PrEP vaccine efficacy against death over 15 years, among individuals not accessing any PEP.

We used a base case value of 0.6, a lower limit of 0.0 and an upper limit of 0.95.

Uncertainty about the appropriate value for this parameter is both high and important for the conclusions of the present analysis. For reasons discussed in the following paragraphs, we used a very broad range of values in our sensitivity analyses, spanning from what we would consider to be an implausibly low lower bound (0.0, i.e. no efficacy) to a very high upper bound (0.95).

There are very few reported cases of PrEP failure in humans [21, 22]. There are, in our view, two likely mechanisms of PrEP efficacy in the absence of PEP.

The first is protection by virus neutralising antibody (VNA) titer ≥0.5 IU/mL at the time of exposure (i.e. pre-formed antibody). It is generally accepted that such titers will be near 100% protective [17, 23, 24]. Efficacy attributable to this mechanism can thus be estimated from available data on VNA kinetics after PrEP. These kinetics depend heavily upon the precise PrEP regime used. Any WHO-recommended PrEP regime will achieve titers exceeding 0.5 IU/mL in >95% of individuals immediately after vaccination. The median duration of maintenance of VNA ≥0.5 IU/mL was <1 year with single-visit PrEP regimes [25-27], while most studies of 3-visit PrEP regimes suggest a median duration of at least 1 year (the latest timepoint in most studies) and in some cases over a decade [28-30]. For regimes achieving a duration of maintenance of VNA ≥0.5 approaching a decade, average PrEP efficacy *attributable to pre-formed VNA alone* could be over 50% over 15 years, but would be lower for 1 or 2 dose schedules.

The second likely mechanism of protection by PrEP, in the absence of PEP, is an anamnestic (recall) response by vaccine-induced memory B cells, induced by the rabies virus itself upon exposure and early infection (probably outside the central nervous system). Vaccine efficacy attributable to exposure-induced recall is hard to estimate as there is no accepted immunological surrogate of protection by this mechanism. We considered three possible strands of evidence in our evaluation of the plausible range for such efficacy: evidence that PrEP will reliably create B cell memory cells that are likely to deliver a rapid recall response for at least 15 years; data demonstrating that recall responses can achieve high levels of protection in the absence of pre-formed antibody in animal challenge models; and evidence from mass PrEP campaigns which are consistent with human PrEP efficacy higher than likely to be explained by pre-formed antibody alone.

There is good evidence that memory B cells induced by other vaccines mediate strong recall responses and persist for life in humans[31], and that a rapid and robust anamnestic response can be induced upon exposure to rabies glycoprotein (in the form of simulated post-exposure vaccination in clinical trials) for at least 15 years after PrEP [28-30].

Studies in animals which had received PrEP at least 1 year before a stringent rabies challenge have shown substantial protection (reviewed by Aubert [32]) [33-35]. Substantial protection persists for at least five years after vaccination in cats and dogs and, importantly, remains even after VNA falls below 0.5 IU/mL. While the strongest protection is associated with higher levels of VNA at the time of challenge, there is significant protection seen at lower or even undetectable (<0.03 IU/mL) VNA levels [32, 33]. 72% of 492 dogs and 83% of 242 cats with undetectable levels of VNA survived challenge 1-year post-vaccination, versus 0% and 20% of unvaccinated controls respectively. Even higher levels of protection were observed in animals with low but detectable VNA (i.e. 0.03<VNA<0.5 IU/mL) [32, 33]. High rates of rabies infection in unvaccinated animals in such studies is achieved through use of high doses of challenge virus (many times the LD_50_) and a route of administration (typically into temporalis muscle) which results in a relatively short incubation period – i.e. a ‘worst case’ exposure scenario.

Finally, data from mass PrEP campaigns in the Philippines and Peru is compatible with very high efficacy over periods of more than two years. PrEP was rolled out in some remote areas of the Amazonas region of Peru where bat rabies was a significant risk (and PEP access was problematic) in 2011 [36, 37]. In 2012 there were only 2 cases of rabies, both in individuals who had refused PrEP, down from 13 in 2010 and 20 in 2011. Other rabies endemic regions that were not included in the PrEP programme showed no decrease in rabies cases. From 2013 to 2017, there were no reported rabies cases in the original region of the PrEP programme [36, 37]. However, the programme was not continued, and in 2024 two death from rabies were reported in the Amazonas region. Both were in children who were too young to have received PrEP while the campaign was last active in their area, in 2013 [38, 39]. This, alongside direct evidence of rabies circulating in wild animals [38, 40], confirm rabies exposure risk remains in the region, yet none of those vaccinated in campaigns over 10 years ago is known to have contracted rabies. Although the 3-dose (days 0, 7, 28) IM PrEP regime used [36] has been shown to be effective at maintaining VNA ≥0.5 IU/mL for at least one year in most and several years in some recipients, a significant number of individuals would be expected not to have maintained this level of VNA [28-30]. It is plausible ongoing protection in this population could be attributable to VNA at levels below 0.5 IU/mL, or exposure-induced recall. In the Philippines, there were no human rabies cases in the trial province of Camarines Sur for two years after implementation of childhood rabies PrEP, although expansion of dog vaccination and access to PEP also contributed to this [41].

We note also that average PrEP efficacy over a 15-year period does not necessarily mean efficacy in the absence of any boosting. A population-wide PrEP approach could conceivably include a booster dose given in later childhood, for example co-administration with HPV vaccine which is commonly given at around 9 years of age.

When these strands of data and reasoning are considered together, we believe it is reasonable to consider the possibility of PrEP efficacy in the likely range of 30% to 80% and less likely range of 0% to 95% over a 15-year period.

#### Discount rate

Discount rates of 0%, 1.5% and 3% per annum were applied to the value of future health in the decision tree model. As the only direct costs in this model are of the delivery of PrEP at the start, the discount rate only applies to the value of future health (ICER per QALY gained) but not consumption of resources. The main results reported in this paper are for a 0% discount rate, with the effect of 1.5% and 3% discount rates on the ICER range in one-way sensitivity analyses and multi-variable contour plots show in Supplementary Figures 1, 2 and 3.

The most recent WHO guide for standardization of economic evaluations of immunization programmes [42] recommends using future health discounts rates of 0% and 3%. We have applied both of these, plus a mid-range rate of 1.5%.

## Calculation of parameters for simplified model ‘base case’

Central values of decision-tree model parameters, as stated in the main paper text (Table 1), were used to calculate simplified model parameters for a ‘base case’ and hence to directly compare the output of the two models. For this comparison, cost of PrEP was assumed to be USD 5 for both models.

The simplified model ‘base case’ used a ‘rabies burden’ (without PrEP) value of 3.021 deaths per 100,000 people per year. This was calculated from central values of decision tree parameters for the probability of being bitten (P_bite_; 0.001), the probability a biting animal is rabid (P_rabid_; 0.3), the probability of being infected with rabies if bitten by a rabid animal (P_infect_; 0.19), the probability of starting PEP within 2 weeks of an exposure (P_startPEP_; 0.5), and the probability PEP prevents rabies in the absence of PrEP (P_prevent1_; 0.94):

$$rabies burden (without PrEP)$$

= [(P_bite_ × P_rabid_ × P_infect_ × (1–P_startPEP_)) + (P_bite_ × P_rabid_ × P_infect_ × P_startPEP_ × (1–P_prevent1_)] × 100 000

$$=\left[ \left( 0.001\times0.3\times0.19\times\left( 1-0.5 \right) \right)+\left( 0.001\times0.3\times0.19\times0.5\times\left( 1-0.94 \right) \right) \right]\times100 000$$

$$=3.021$$

The simplified model ‘base case’ used a PrEP efficacy of 0.623, which was calculated from the proportion of lives saved under the rabies burden with PrEP scenario vs the rabies burden without PrEP scenario. For those who receive PrEP, the rabies burden is altered through lives saved amongst those not accessing PEP (P_prevent3_; 0.6) and improvement in the survival of those who also received PEP (P_prevent2_; 1.0):

$$rabies burden (with PrEP)$$

= [(P_bite_ × P_rabid_ × P_infect_ × (1–P_startPEP_) × (1–P_prevent3_))+(P_bite_ × P_rabid_ × P_infect_ × P_startPEP_ × (1–P_prevent2_)] × 100 000

$$=\left[ \left( 0.001\times0.3\times0.19\times\left( 1-0.5 \right)\times(1-0.6 \right)+\left( 0.001\times0.3\times0.19\times0.5\times\left( 1-1.0 \right) \right) \right]\times100 000$$

$$=1.14$$

$$PrEP efficacy$$

$$=\frac{rabies burden \left( without PrEP \right)-rabies budren (with PrEP)}{rabies burden (without PrEP)}$$

$$=\frac{3.021-1.14}{3.021}$$

$$=0.623$$

# Supplementary figures

Supplementary Figure 1. Tornado plot of one-way sensitivity analysis of key parameters using the decision tree model when a 0% discount rate (A), 1.5% discount rate (B), or 3% discount rate (C) was applied. Part (A) is the same information as in Figure 2 in the main paper text. Parameter values used are outlined in Table 1: values of one parameter at a time (indicated on the y axis) were varied within the stated range, while all other parameters were fixed at the base case values. The effect of decreasing and increasing each parameter value on the ICER are indicated by the pink and blue coloured bars respectively. The black vertical lines indicate the baseline scenario under each discounting strategy. The ICER under a no-discount strategy (A) was 354 USD, under a 1.5% discount strategy (B) 506 USD and under a 3% discount strategy (C) 689 USD.


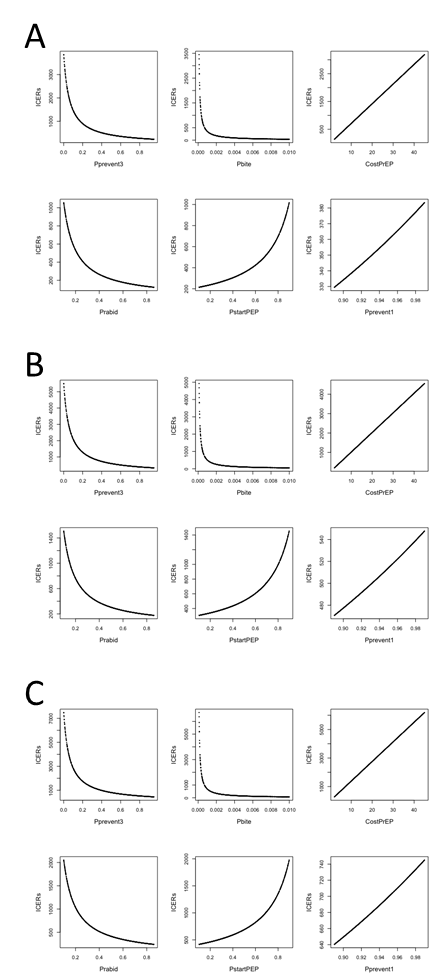


Supplementary Figure 2. Individual plots of the impact of varying each key parameter in a one-way sensitivity analysis when a 0% discount rate (A), 1.5% discount rate (B), or 3% discount rate (C) was applied. Part (A) is the same information as in Figure 3 in the main paper text. Values of the parameter indicated were varied on the x axis within a range and values of other parameter values fixed as outlined in Table 1. Note the scale of the y axis (ICERs) varies between each plot.

B

A

C

Supplementary Figure 3. Contour plots of the ICER (cost in USD to gain one QALY through averting death by rabies) across a range of variables under conditions of a 0% discount rate (A), 1.5% discount rate (B), and 3% discount rate (C). Part (A) is the same information as in Figure 4 in the main paper text. In each grid of 5 × 5 individual contour plots, the probability PrEP prevents rabies without any administration of PEP after an exposure varies between the columns as shown on the outer X-axis, with 0 for the left column and 0.95 or the right column. The cost of rabies PrEP per person in USD varies between the rows as shown on the outer Y-axis, with 45 USD for the top row and the 2 USD for the bottom row. For each individual contour plot, rabies exposure risk per 100 000 people (made up of P_bite_ × P_rabid_ × P_infect_ × 100 000) varies across the X axis from 0.56 to 19, while access to PEP (measured as the probability of starting PEP following a rabies exposure, or P_startPEP_) varies on the Y axis from 0.1 to 0.9. Poor access to PEP (inner Y axis), higher levels of rabies risk (inner x axis), lower cost of PrEP (outer y axis) and higher efficacy of PrEP in preventing rabies in the absence of PEP (outer x axis) all lower the ICER for PrEP.

# Supplementary references

[1] Hampson K, Abela-Ridder B, Bharti O, Knopf L, Léchenne M, Mindekem R, et al. Modelling to inform prophylaxis regimens to prevent human rabies. Vaccine. 2019;37 Suppl 1:A166-a73.

[2] Quiambao B, Varghese L, Demarteau N, Sengson RF, Javier J, Mukherjee P, et al. Health economic assessment of a rabies pre-exposure prophylaxis program compared with post-exposure prophylaxis alone in high-risk age groups in the Philippines. International journal of infectious diseases : IJID : official publication of the International Society for Infectious Diseases. 2020;97:38-46.

[3] Royal A, John D, Bharti O, Tanwar R, Bhagat DK, Padmawati RS, et al. A Cost-Effectiveness Analysis of Pre-Exposure Prophylaxis to Avert Rabies Deaths in School-Aged Children in India. Vaccines (Basel). 2022;11.

[4] Global vaccine market report 2022: a shared understanding for equitable access to vaccines. Geneva: World Health Organisation. 2023.

[5] Joe CCD, Jiang J, Linke T, Li Y, Fedosyuk S, Gupta G, et al. Manufacturing a chimpanzee adenovirus-vectored SARS-CoV-2 vaccine to meet global needs. Biotechnol Bioeng. 2022;119:48-58.

[6] World Health Organization. WHO Expert Consultation on Rabies. Third report. World Health Organization technical report series. 2018:1-184, back cover.

[7] Changalucha J, Steenson R, Grieve E, Cleaveland S, Lembo T, Lushasi K, et al. The need to improve access to rabies post-exposure vaccines: Lessons from Tanzania. Vaccine. 2019;37:A45-A53.

[8] Hampson K, Dobson A, Kaare M, Dushoff J, Magoto M, Sindoya E, et al. Rabies exposures, post-exposure prophylaxis and deaths in a region of endemic canine rabies. PLoS neglected tropical diseases. 2008;2:e339.

[9] Ly S, Buchy P, Heng NY, Ong S, Chhor N, Bourhy H, et al. Rabies situation in Cambodia. PLoS neglected tropical diseases. 2009;3:e511.

[10] Tricou V, Bouscaillou J, Kamba Mebourou E, Koyanongo FD, Nakouné E, Kazanji M. Surveillance of Canine Rabies in the Central African Republic: Impact on Human Health and Molecular Epidemiology. PLoS neglected tropical diseases. 2016;10:e0004433.

[11] Hampson K, Coudeville L, Lembo T, Sambo M, Kieffer A, Attlan M, et al. Estimating the global burden of endemic canine rabies. PLoS Negl Trop Dis. 2015;9:e0003709.

[12] Agarwal N, Singh C, Gulsha K, Singh S, Sinha S, Singh K, et al. Epidemiology of Dog Bites in Patna: A Cross Sectional Study. Indian Journal of Community and Family Medicine. 2015;1:70-4.

[13] Kapur V. Is Pre-Exposure Prophylaxis a Cost-Effective Intervention to Avert Rabies Deaths among School-Aged Children in India? Comment on Royal et al. A Cost-Effectiveness Analysis of Pre-Exposure Prophylaxis to Avert Rabies Deaths in School-Aged Children in India. Vaccines 2023, 11, 88. Vaccines (Basel). 2023;11.

[14] WHO Rabies Modeling Consortium. The potential effect of improved provision of rabies post-exposure prophylaxis in Gavi-eligible countries: a modelling study. The Lancet Infectious diseases. 2019;19:102-11.

[15] Lembo T, Hampson K, Haydon DT, Craft M, Dobson A, Dushoff J, et al. Exploring reservoir dynamics: a case study of rabies in the Serengeti ecosystem. J Appl Ecol. 2008;45:1246-57.

[16] Shim E, Hampson K, Cleaveland S, Galvani AP. Evaluating the cost-effectiveness of rabies post-exposure prophylaxis: A case study in Tanzania. Vaccine. 2009;27:7167-72.

[17] World Health Organization. Rabies vaccines: WHO position paper, April 2018 - Recommendations. Vaccine. 2018;36:5500-3.

[18] Shantavasinkul P, Tantawichien T, Wacharapluesadee S, Jeamanukoolkit A, Udomchaisakul P, Chattranukulchai P, et al. Failure of rabies postexposure prophylaxis in patients presenting with unusual manifestations. Clin Infect Dis. 2010;50:77-9.

[19] Tinsa F, Borgi A, Jahouat I, Boussetta K. Rabies encephalitis in a child: a failure of rabies post exposure prophylaxis? BMJ Case Rep. 2015;2015.

[20] Wilde H. Failures of post-exposure rabies prophylaxis. Vaccine. 2007;25:7605-9.

[21] Lodha L, Manoor Ananda A, Mani RS. Rabies control in high-burden countries: role of universal pre-exposure immunization. Lancet Reg Health Southeast Asia. 2023;19:100258.

[22] Bernard KW, Fishbein DB, Miller KD, Parker RA, Waterman S, Sumner JW, et al. Pre-exposure rabies immunization with human diploid cell vaccine: decreased antibody responses in persons immunized in developing countries. Am J Trop Med Hyg. 1985;34:633-47.

[23] Rao AK, Briggs D, Moore SM, Whitehill F, Campos-Outcalt D, Morgan RL, et al. Use of a Modified Preexposure Prophylaxis Vaccination Schedule to Prevent Human Rabies: Recommendations of the Advisory Committee on Immunization Practices - United States, 2022. MMWR Morb Mortal Wkly Rep. 2022;71:619-27.

[24] World Health Organization. Rabies: rationale for investing in the global elimination of dog-mediated human rabies. 2015.

[25] Jonker EFF, Visser LG. Single visit rabies pre-exposure priming induces a robust anamnestic antibody response after simulated post-exposure vaccination: results of a dose-finding study. Journal of travel medicine. 2017;24.

[26] Overduin LA, Koopman JPR, Prins C, Verbeek-Menken PH, De Pijper CA, Eblé PL, et al. Boostability after single-visit pre-exposure prophylaxis with rabies vaccine: a randomised controlled non-inferiority trial. The Lancet Infectious diseases. 2024;24:206-16.

[27] Soentjens P, Andries P, Aerssens A, Tsoumanis A, Ravinetto R, Heuninckx W, et al. Preexposure Intradermal Rabies Vaccination: A Noninferiority Trial in Healthy Adults on Shortening the Vaccination Schedule From 28 to 7 Days. Clin Infect Dis. 2019;68:607-14.

[28] De Pijper CA, Langedijk AC, Terryn S, Van Gucht S, Grobusch MP, Goorhuis A, et al. Long-term Memory Response After a Single Intramuscular Rabies Booster Vaccination 10-24 Years After Primary Immunization. J Infect Dis. 2022;226:1052-6.

[29] Suwansrinon K, Wilde H, Benjavongkulchai M, Banjongkasaena U, Lertjarutorn S, Boonchang S, et al. Survival of neutralizing antibody in previously rabies vaccinated subjects: a prospective study showing long lasting immunity. Vaccine. 2006;24:3878-80.

[30] Langedijk AC, De Pijper CA, Spijker R, Holman R, Grobusch MP, Stijnis C. Rabies Antibody Response After Booster Immunization: A Systematic Review and Meta-analysis. Clin Infect Dis. 2018;67:1932-47.

[31] Crotty S, Felgner P, Davies H, Glidewell J, Villarreal L, Ahmed R. Cutting edge: long-term B cell memory in humans after smallpox vaccination. J Immunol. 2003;171:4969-73.

[32] Aubert MF. Practical significance of rabies antibodies in cats and dogs. Rev Sci Tech. 1992;11:735-60.

[33] Bunn TO. Cat rabies. In: Baer GM, editor. The natural history of rabies, 2nd Ed: CRC Press; 1991. p. 379-87.

[34] Dodds WJ, Larson LJ, Christine KL, Schultz RD. Duration of immunity after rabies vaccination in dogs: The Rabies Challenge Fund research study. Can J Vet Res. 2020;84:153-8.

[35] Zhang X. Evaluation of long-term protective efficacy of rabies vaccines in dogs, MSc thesis. Athens, Georgia: The University of Georgia; <https://getd.libs.uga.edu/pdfs/zhang_xiwen_201608_ms.pdf>, accessed 23 July 2024. 2016.

[36] Recuenco SE. Rabies Vaccines, Prophylactic, Peru: Massive Rabies Pre-exposure Prophylaxis for High-Risk Populations. In: Ertl HCJ, editor. Rabies and Rabies Vaccines. Cham: Springer International Publishing; 2020. p. 83-101.

[37] Rupprecht CE, Mani RS, Mshelbwala PP, Recuenco SE, Ward MP. Rabies in the Tropics. Curr Trop Med Rep. 2022;9:28-39.

[38] National Center for Epidemiology Prevention and Disease Control (Peru). Rabies, <https://www.dge.gob.pe/portal/docs/vigilancia/sala/2024/SE08/rabia.pdf>, accessed 08 July 2024. 2024.

[39] Recuenco S. Presonal communication: Rabies PrEP in Peru update. 03 April 2024.

[40] National Center for Epidemiology Prevention and Disease Control (Peru). Rabies Surveillance, Prevention and Control. <https://www.dge.gob.pe/portalnuevo/vigilancia-epidemiologica/vigilancia-de-enfermedades-zoonoticas/#tab-content-4>. Accessed 08 July 2024. 2024.

[41] Dodet B. Report of the sixth AREB meeting, Manila, The Philippines, 10-12 November 2009. Vaccine. 2010;28:3265-8.

[42] WHO guide for standardization of economic evaluations of immunization programmes, 2nd edition. Geneva: World Helath Organization. 2019.
